# Inflammation of the Os and Cervix Uteri

**Published:** 1856-03

**Authors:** 


### RECORD OF MEDICAL SCIENCE.

*Inflammation of the Os and Cervix Uteri.* By Dr. Rigby.

Besides arising from general derangement of the system, and an unhealthy condition of the circulation, inflammation of the os and cervix uteri may be induced by causes of a mere local character. Thus, for instance, exposure to cold, but this is more frequently seen to produce inflammation of the ovaries, particularly if acting during a catamenial period; violent horse exercise, especially if, from want of healthy tone, the uterus has descended lower than usual into the pelvis; inordinate or unhealthy sexual intercourse; and improper irritation of the part.

It will be scarcely necessary for me to dwell on cases of this sort, but there is another cause, which would, perhaps, come under the last head, which has existed of late years to an incredible extent, and which has not only produced much local mischief, but, in many cases, has caused a serious amount of spinal irritation, attended with severe suffering, shattered health, and forming a train of constitutional and local symptoms, the treatment of which is both difficult and tedious. I allude to the dishonest application of caustic to the os uteri, at intervals so short, as to render it impossible that the eschar produced by the first application should have healed before the next was made, and continued at this rate for such a length of time as to set up severe irritation, and even produce serious injury.

This form of inflammation of the os and cervix, presents features which distinguish it from the ordinary species I have been describing. It would, perhaps, be correct to call it a highly irritable condition of the uterus (which not only involves the ovaries, but frequently implicates the spinal cord to a serious extent,) were it not for the actual alteration of structure and permanent injury of the part which has suffered more immediately from this treatment.

The patient complains of constant pain in the uterine region, but generally more behind the symphysis pubis than is usually the case in ordinary inflammation of the cervix, extending from hip to hip, and in aggravated cases darting up the spine with neuralgic severity. It is increased by standing or by any exercise whatever. Sitting down upon a hard seat, or the passage down the rectum of solid fæces, produces great suffering, inasmuch as when once the pain is brought on, it will continue severely for some time after. The moment she assumes the erect posture she has a sensation of weight, bearing down, and burning heat in the pelvis, which are quickly followed by the pain itself.

The catamenial periods usually follow each other too quickly; are almost always very profuse, and invariably attended with great suffering. If the ovaries are involved, the symptoms of ovarian dysmenorrhœa will also be present. She has a constant ichorous watery discharge, which is sometimes very profuse. The bowels are unhealthy; the urine thick; the tongue pale, dry, and rough, with red papillæ; the face is sallow and wan; the spirits depressed; the pulse feeble and very irritable; and she has lost strength and flesh.

On examination, per vaginam, this canal is found soaked in the thin, watery discharge already mentioned. On gently touching the os and cervix uteri with the finger, the patient feels as if the parts were raw, from the aggravated sensibility which now exists in them; firmer pressure with the finger brings on the neuralgic pain already described. The os uteri is usually swollen, uneven, and knobby—it is frequently dragged forwards, or to one side, without any corresponding displacement of the organ itself. Whether this alteration of shape is owing to cicatrization in the part itself, and surrounding vagina; or whether it depends on different portions being affected with different degrees of induration, it is not easy to determine; at any rate the cervix usually has a strong degree of hardness, and the uterus above it feels large, hard, and very tender to the touch.

Seen through the speculum, the os uteri does not present the dark red tinge, more or less mottled with patches of a brighter color, as in cases of ordinary inflammation, but it has a pale ashy hue much injected with vessels, just as is occasionally seen in the irritable throat and tonsils of an unhealthy person who has been suffering from repeated attacks of quinsy.

The discharge is evidently uterine, and is constantly oozing from the os uteri in considerable quantities. If the uterine sound be passed, it generally penetrates half-an-inch, or even a whole inch, beyond the usual distance, showing that the uterine cavity is enlarged; and intense pain is produced the instant it touches the internal surface of the uterus, indicating great irritability of that organ, and probably inflammation of its lining membrane.

The treatment of this affection will necessarily differ a good deal from the ordinary forms of inflammation of the os and cervix; the indications, it is true, will, to a certain extent, be the same, but the local as well as the general condition of the patient is very different. The inflammation of the os and cervix in these cases is seldom of such a nature as to require the application of leeches to the part itself, and the extreme irritation which they not unfrequently produce contra-indicates any local applications but those of a soothing character; while the enfeebled state of the patient's health renders any depletion very questionable.

The general treatment is by no means so simple or so easy as in the other case. The health has been so much deranged by the long-continued effects of severe uterine irritation, and the strength so broken down by frequent attacks of menorrhagia, and the profuse leucorrhœa during the intervals, that it is difficult to adopt any distinct line of treatment at first starting, beyond the attempt to regulate the liver and bowels by the mildest remedies, and soothe the irritable system by gentle sedatives.

A course of taraxacum, with decoct. sarzæ comp. and liq. calcis, is valuable in the early stage of treatment, as it obviates the necessity of mercurial alteratives, which are usually contra-indicated by the mucous irritation of the intestinal canal. It is of great importance to allay this condition as quickly as possible, not only because we thereby remove a fruitful source of uterine irritation, but because it also enables us to employ remedies which we could not otherwise do. When this has been arrested, we may generally pass at once to the use of mineral acids and tonics, and, if necessary, these will pave the way to a course of cod-liver oil. If the season of the year permit, a residence for some months at the sea-side will be most desirable. The patient should use sea-water in her morning ablutions, and, if her health be sufficiently improved, and the weather warm enough, she may bathe in the sea with advantage.

The local treatment, as I have before stated, must be essentially of a soothing character. Liq. plumbi diacet. in decoct. papaveris forms one of the best injections where there is any amount of heat, swelling, or discharge; in other cases, the plain poppy decoction appears to be the only local application which will give relief. As it is desirable she should retain injections of this sort for fifteen or twenty minutes, she ought to lie upon her back, with her heels drawn up to the nates, and thus give the vagina such a direction that the injection will remain in it as long as she preserves this position. The suppositories of diacetate of lead and extract of conium are also very valuable in these cases, and, by being in a more concentrated form, and capable of being retained much longer, produce a much greater effect. The warm hip-bath is very useful, and with some patients, appears to be the only thing which gives relief. If, however, the weather be mild, the sooner she can gradually come to the use of cold water the better; and she may always be looked upon as having made good progress when, during her residence at the sea-side, she is able to bear cold sponging with sea-water, and bathing in the open sea. For those who are unable to enjoy these advantages, the salt towel which I have before recommended will be an excellent adjunct to the cold sponging. But it will be needless to enter into further details; they will vary more or less with every patient, or with the same patient at different times, and will be naturally suggested by the peculiar features, etc. of each case. The indications of treatment are to allay irritation, and restore tone, but the treatment itself must necessarily be modified by the circumstances of each particular case.

I am sorry to acknowledge that I find no difficulty in illustrating this, in many respects, disreputable subject; for my supply of cases, of which I have taken notes, is very considerable. In 1847 and 1848, when the speculum furor was rising to its height, they were few and far between; but during the last three or four years, the frequency of these cases has greatly increased. The dishonest use of the speculum and caustic is gradually working its own cure, not, alas! upon the perpetrators, but many a miserable broken-down invalid, to whom life has become a state of continued suffering, bitterly rues the day when the overdrawn statements of some gossiping friend, or her own love of "what is the fashion," prevailed on her credulity to entrust herself into the hands of these unscrupulous members of our Profession.

As regards cases for illustrating the foregoing remarks, the only difficulty is in the selection and arrangement of them; for, as I have before stated, the supply is abundant. I will begin with some of the milder cases among married women :—

Mrs. A., aged 23, married three months.

January 5.—Delicate, just recovered from low fever with influenza, much headache; had slight swelling and tenderness of the lower part of the abdomen, on the left side, which has been leeched with relief; is of a constipated habit. No leucorrhœa.

Three weeks ago, was under the treatment of one who has not deemed it inconsistent with his creed to adopt this practice :—he had applied caustic to the os uteri three times, with intervals of only four days, on account, as he said, of hardness of the cervix.

*Examination per vaginam.*—The os and uteri is turned rather forwards, but I do not detect the slightest indication of the fundus backwards. The os and cervix, when touched, feel sore, as if raw, which she says was never the case until the caustic was applied.

Mistura ferri et magnesiæ sulph. o. m., Acid. nitro-muriat. c. tarax-aco ex infus. gentianæ co., ter die; Suppositorium plumbi diacet. c. extr. conii o. n.

14.—Much better.

*Examination cum speculo.*—Os uteri quite healthy; rep. med.

March 25.—Is quite well, and has every reason to suppose herself pregnant.

The details of this patient's previous history are scanty and imperfect; but it may be safely inferred, that the attack of inflammatory pain in the vicinity of the sigmoid flexure of the colon was probably a result of the constipation to which she was liable.

It seems rather strange that hardness of the cervix should be considered as indicating the application of solid nitrate of silver! No practitioner would think of applying it in this way to any other glandular tissue in a state of irritable induration, except, perhaps, the homœopathists, who, by such treatment, would illustrate their celebrated dictum of "similia similibus curantur." In the present case it was much the same, the practitioner having applied an irritant to relieve irritation!

The lunar caustic was applied but three times, but at intervals so short, that the raw surface thus produced could not possibly have been healed over; in fact, even the eschar of dead epithelium could not have been thrown off much more than twenty-four hours before it was applied again, so that the second and third applications of caustic were evidently made to a raw surface. If a blister were applied every fourth day to the same spot on the external surface of the body, we should naturally expect to produce in a short time an ugly ulceration, of a highly irritable character; and if the part were in the neighborhood of superficial, or rather glandular structures, we should naturally expect to find them soon becoming hard and tender, as a necessary result of irritation thus fiercely applied; and yet, when directly applied to an internal and highly irritable glandular tissue, the greater part of which is covered only by a delicate mucous membrane, and this application frequently repeated at such short intervals, that the surface is entirely, and sometimes deeply, denuded of its natural covering, we are to suppose that such treatment will not only produce no irritation, but even remove an existing state of irritation, and render a glandular tissue soft, which has become hard from irritation. Such treatment applied to an irritable breast or testicle, especially where the habit of body is feeble, and the health deranged, would reasonably lead us to expect, sooner or later, the establishment of organic disease. Another effect of this treatment, which I shall have occasion to point out in more aggravated cases, was slightly manifested in the present instance; I allude to a distorted condition of the os uteri, apparently the result of unequal contraction of the lower part of the cervix, so that the os is dragged out of its usual direction. In this case, finding it turned somewhat forwards, I was reminded of the similar condition in common retroversion of the unimpregnated uterus, and feel convinced that it was an effect of the hardness of the cervix, arising from the irritation of the lunar caustic. In worse cases it is permanent, being produced by deep cicatrizations not only of the os and cervix, but of the upper part of the vagina; in slight cases, like the one under consideration, it would disappear as the part returned to its natural condition. I regret that I did not specially notice this point at my last examination.

One other result I must not omit, as I shall have frequent occasion to allude to it when existing in a far greater degree, and which, in this patient, was only to a trifling extent, viz: the sense of soreness and rawness of the os and cervix when touched. Three weeks had elapsed since the last application of caustic, and she had no leucorrhœa, so that there was every reason to suppose that the part was quite healed over; nor is this smarting sensation necessarily produced by passing the finger over a part to which caustic has been recently applied—it is a slight and early manifestation of that morbid sensibility which, in its more severe forms, is marked by those paroxysms of pelvic pain and neuralgic darts of acute suffering, which seem to have their seat in the more immediate vicinity of the spinal cord.

The treatment was according to the indications which I have stated, viz: to strengthen the general health, and soothe local irritation; and was followed with the best results.—*Medical Times and Gazette.*

